# [2,7-Dimeth­oxy-8-(4-meth­oxy­benzo­yl)naphthalen-1-yl](4-meth­oxy­phen­yl)methanone chloro­form monosolvate

**DOI:** 10.1107/S1600536812050799

**Published:** 2012-12-22

**Authors:** Kosuke Sasagawa, Rei Sakamoto, Taro Kusakabe, Akiko Okamoto, Noriyuki Yonezawa

**Affiliations:** a Department of Organic and Polymer Materials Chemistry, Tokyo University of Agriculture & Technology, Koganei, Tokyo 184-8588, Japan

## Abstract

In the title compound, C_28_H_24_O_6_·CHCl_3_, the two 4-meth­oxy­benzoyl groups at the 1- and 8-positions of the naphthalene ring system are aligned almost anti­parallel, the benzene rings making a dihedral angle of 25.76 (7)°. The naphthalene ring system makes dihedral angles of 72.51 (7) and 73.33 (7)° with the benzene rings. In the crystal, the naphthalene mol­ecules are linked by C—H⋯O inter­actions, forming a helical chain along the *b-*axis direction. A C—H⋯Cl inter­action is also observed between the aroylated naphthalene and chloro­form mol­ecules. The chloro­form mol­ecule is disordered over two positions with site occupancies of 0.478 (5) and 0.522 (5).

## Related literature

For the formation reaction of aroylated naphthalene compounds *via* electrophilic aromatic substitution of naphthalene derivatives, see: Okamoto *et al.* (2011 ▶); Okamoto & Yonezawa (2009 ▶). For structures of closely related compounds, see: Hijikata *et al.* (2010 ▶); Sasagawa *et al.* (2011 ▶).
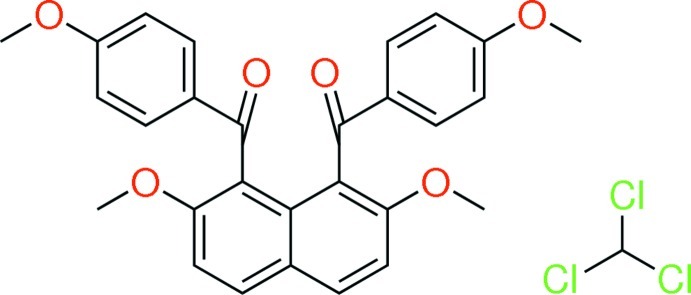


## Experimental

### 

#### Crystal data

C_28_H_24_O_6_·CHCl_3_
*M* *_r_* = 575.84
Monoclinic, 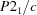

*a* = 8.2781 (2) Å
*b* = 21.4763 (5) Å
*c* = 15.5370 (4) Å
β = 98.448 (2)°
*V* = 2732.25 (12) Å^3^
*Z* = 4
Cu *K*α radiation
μ = 3.39 mm^−1^
*T* = 193 K
0.50 × 0.20 × 0.10 mm

#### Data collection

Rigaku R-AXIS RAPID diffractometer
Absorption correction: numerical (*NUMABS*; Higashi, 1999 ▶) *T*_min_ = 0.282, *T*_max_ = 0.728
50703 measured reflections
4994 independent reflections
4305 reflections with *I* > 2σ(*I*)
*R*_int_ = 0.047

#### Refinement

*R*[*F* ^2^ > 2σ(*F* ^2^)] = 0.035
*wR*(*F* ^2^) = 0.107
*S* = 1.10
4994 reflections
385 parameters
H-atom parameters constrained
Δρ_max_ = 0.24 e Å^−3^
Δρ_min_ = −0.35 e Å^−3^

### 

Data collection: *PROCESS-AUTO* (Rigaku, 1998 ▶); cell refinement: *PROCESS-AUTO*; data reduction: *CrystalStructure* (Rigaku, 2010 ▶); program(s) used to solve structure: *SHELXS97* (Sheldrick, 2008 ▶); program(s) used to refine structure: *SHELXL97* (Sheldrick, 2008 ▶); molecular graphics: *ORTEPIII* (Burnett & Johnson, 1996 ▶); software used to prepare material for publication: *SHELXL97*.

## Supplementary Material

Crystal structure: contains datablock(s) I, global. DOI: 10.1107/S1600536812050799/is5230sup1.cif

Structure factors: contains datablock(s) I. DOI: 10.1107/S1600536812050799/is5230Isup2.hkl

Supplementary material file. DOI: 10.1107/S1600536812050799/is5230Isup3.cml

Additional supplementary materials:  crystallographic information; 3D view; checkCIF report

## Figures and Tables

**Table 1:** Hydrogen-bond geometry (Å, °)

*D*—H⋯*A*	*D*—H	H⋯*A*	*D*⋯*A*	*D*—H⋯*A*
C7—H7⋯O5^i^	0.95	2.37	3.1460 (19)	139
C13—H13⋯Cl3^ii^	0.95	2.75	3.647 (2)	159

Symmetry codes: (i) 
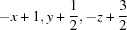
; (ii) 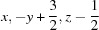.

**Fig. 1.**
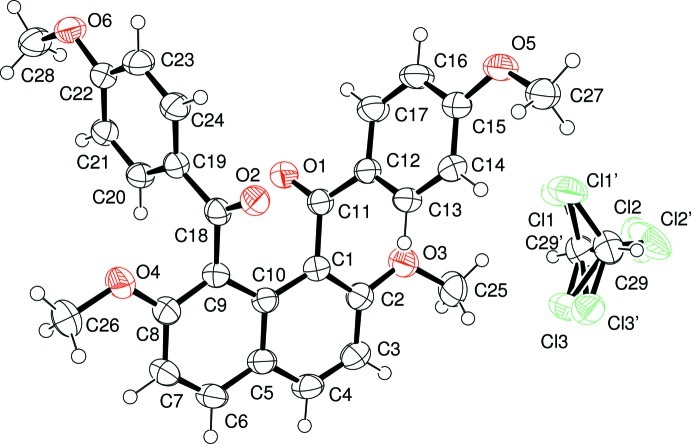
Molecular structure with the atom-labeling scheme and displacement ellipsoids drawn at the 50% probability level. Hydrogen atoms are shown as small spheres of arbitrary radius.

**Fig. 2.**
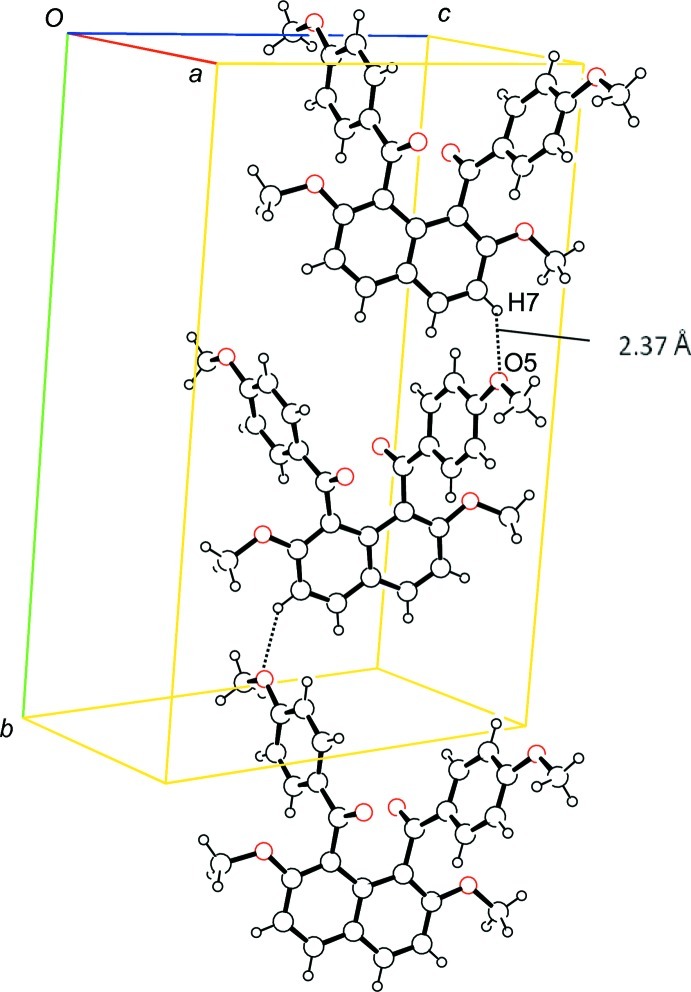
Intermolecular C—H···O interactions between atoms H7 and O5 [symmetry code: x, 3/2 - y, -1/2 + z] along the b axis (dashed lines).

**Fig. 3.**
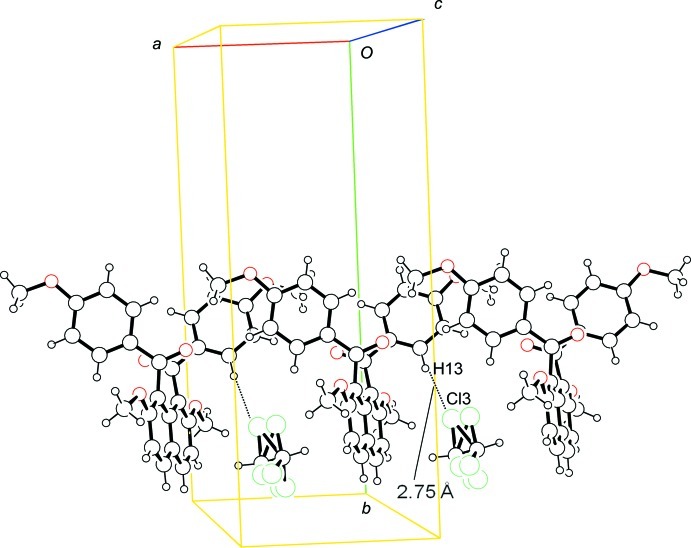
Intermolecular C—H···Cl interactions between atoms H13 and Cl3 [symmetry code: 1 - x, 1/2 + y, 3/2 - z] along the a axis (dashed lines).

## supplementary crystallographic information

### Comment

In the course of our study on selective electrophilic aromatic aroylation of the naphthalene ring core, 1,8-diaroylnaphthalene compounds have proved to be formed regioselectively by the aid of a suitable acidic mediator (Okamoto & Yonezawa, 2009; Okamoto *et al.*, 2011). Recently, we have reported the X-ray crystal structures of 1,8-diaroylated 2,7-dimethoxynaphthalene derivatives such as {8-[4-(butoxy)benzoyl]-2,7-dimethoxynaphthalen-1-yl}[4-(butoxy)phenyl]methanone [1,8-bis(4-butoxylbenzoyl)-2,7-dimethoxynaphthalene] (Sasagawa *et al.*, 2011).

The aroyl groups in these compounds are almost perpendicular to the naphthalene rings, and are oriented in opposite directions (*anti*-orientation). On the other hand, we have also clarified minor structure of 1,8-diaroylnaphthalene derivatives, which the aroyl groups are oriented in same direction (*syn*-orientation) [2,7-dimethoxy-1,8-bis(4-phenoxybenzoyl)naphthalene; Hijikata *et al.*, 2010]. As a part of our ongoing studies on the molecular structures of these kinds of homologous molecules, the X-ray crystal structure of the title compound, 1,8-diaroylated naphthalene bearing methoxy groups on the aroyl groups, is discussed in this article.

The molecular structure of the title compound is displayed in Fig. 1. Two 4-methoxybenzoyl groups are situated in the *anti*-orientation. The dihedral angle between the best planes of the two phenyl rings is 25.76 (7)°. The dihedral angles between the best planes of the two 4-methoxyphenyl rings and the naphthalene ring are 72.51 (7) and 73.33 (7)°, respectively. The dihedral angles between the naphthalene ring system and the bridging ketonic carbonyl C—C(═O)—C planes [65.95 (8) and 68.67 (6)°] are larger than those between the phenyl rings and the bridging carbonyl planes [8.40 (8) and 5.49 (7)°].

In the molecular packing, C—H···O interactions between the ethereal oxygen atom of the benzene and the aromatic hydrogen atom of the naphthalene are observed. The C—H···O interactions effectively contribute to stabilization of the molecular packing (C7—H7···O5 = 2.37 Å; symmetry code: *x*, 3/2 - *y*, -1/2 + *z*; Fig. 2). Furthermore, chloroform solvent molecules lie between two naphthalene rings, and form meaningful C—H···Cl interactions with the aromatic hydrogen atoms of benzene rings (C13—H13···Cl3 = 2.75 Å; symmetry code: 1 - *x*, 1/2 + *y*, 3/2 - *z*; Fig. 3).

### Experimental

4-Methoxylbenzoyl chloride (6.60 mmol, 1.13 g), titanium chloride (19.8 mmol, 3.76 g) and methylene chloride (5.00 ml) were placed into a 50 ml flask, followed by stirring at room temperature. To the reaction mixture thus obtained, 2,7-dimethoxynaphthalene (2.00 mmol, 376 mg) was added. The reaction mixture was poured into ice-cold water (100 ml) after it had been stirred for 6 h at room temperature. The aqueous layer was extracted with CHCl_3_ (20 ml × 3). The combined extracts were washed with 2 *M* aqueous NaOH followed by washing with brine. The extracts thus obtained were dried over anhydrous MgSO_4_. The solvent was removed under reduced pressure to give a cake. The crude product was purified by recrystallization from hexane and CHCl_3_ (yield 45%). Colorless platelet single crystals suitable for X-ray diffraction were obtained by repeated crystallization from CHCl_3_.

Spectroscopic Data:

^1^H NMR *δ* (300 MHz, CDCl_3_): 3.70 (6*H*, s), 3.83 (6*H*, s), 6.80 (4*H*, broad), 7.15 (2*H*, d, *J* = 9.0 Hz), 7.64 (4*H*, broad), 7.92 (2*H*, d, *J* = 9.0 Hz). ^13^C NMR δ (75 MHz, CDCl_3_): 55.24, 56.49, 111.24, 113.20, 121.89, 125.54, 129.62, 131.39, 131.71, 132.05, 155.93, 163.03, 194.98 p.p.m.. IR (KBr): 1651 (C=O), 1600, 1575, 1508 (Ar), 1250 (OMe) cm^-1^. HRMS (m/*z*): [*M*+H]^+^ calcd. for C_28_H_25_O_6,_ 457.1651, found, 457.1691. m.p. = 475.9–476.9 K

### Refinement

All H atoms were found in a difference map and were subsequently refined as riding atoms, with C—H = 0.95 (aromatic), 0.98 (methyl) and 1.00 (chloroform) Å, and with *U*_iso_(H) = 1.2*U*_eq_(C).

### Crystal data

**Table:**

C_28_H_24_O_6_·CHCl_3_	*F*(000) = 1192
*M**_r_* = 575.84	*D*_x_ = 1.400 Mg m^−^^3^
Monoclinic, *P*2_1_/*c*	Cu *K*α radiation, λ = 1.54187 Å
Hall symbol: -P 2ybc	Cell parameters from 37515 reflections
*a* = 8.2781 (2) Å	θ = 3.5–68.2°
*b* = 21.4763 (5) Å	µ = 3.39 mm^−^^1^
*c* = 15.5370 (4) Å	*T* = 193 K
β = 98.448 (2)°	Platelet, colorless
*V* = 2732.25 (12) Å^3^	0.50 × 0.20 × 0.10 mm
*Z* = 4	

### Data collection

**Table:**

Rigaku R-AXIS RAPID diffractometer	4994 independent reflections
Radiation source: fine-focus sealed tube	4305 reflections with *I* > 2σ(*I*)
Graphite monochromator	*R*_int_ = 0.047
Detector resolution: 10.000 pixels mm^-1^	θ_max_ = 68.2°, θ_min_ = 3.5°
ω scans	*h* = −9→9
Absorption correction: numerical (*NUMABS*; Higashi, 1999)	*k* = −25→25
*T*_min_ = 0.282, *T*_max_ = 0.728	*l* = −18→18
50703 measured reflections	

### Refinement

**Table:**

Refinement on *F*^2^	Secondary atom site location: difference Fourier map
Least-squares matrix: full	Hydrogen site location: inferred from neighbouring sites
*R*[*F*^2^ > 2σ(*F*^2^)] = 0.035	H-atom parameters constrained
*wR*(*F*^2^) = 0.107	*w* = 1/[σ^2^(*F*_o_^2^) + (0.0633*P*)^2^ + 0.2582*P*] where *P* = (*F*_o_^2^ + 2*F*_c_^2^)/3
*S* = 1.10	(Δ/σ)_max_ = 0.004
4994 reflections	Δρ_max_ = 0.24 e Å^−^^3^
385 parameters	Δρ_min_ = −0.35 e Å^−^^3^
0 restraints	Extinction correction: *SHELXL97* (Sheldrick, 2008), Fc^*^=kFc[1+0.001xFc^2^λ^3^/sin(2θ)]^-1/4^
Primary atom site location: structure-invariant direct methods	Extinction coefficient: 0.00145 (17)

### Special details

**Table:**

Geometry. All e.s.d.'s (except the e.s.d. in the dihedral angle between two l.s. planes) are estimated using the full covariance matrix. The cell e.s.d.'s are taken into account individually in the estimation of e.s.d.'s in distances, angles and torsion angles; correlations between e.s.d.'s in cell parameters are only used when they are defined by crystal symmetry. An approximate (isotropic) treatment of cell e.s.d.'s is used for estimating e.s.d.'s involving l.s. planes.
Refinement. Refinement of *F*^2^ against ALL reflections. The weighted *R*-factor *wR* and goodness of fit *S* are based on *F*^2^, conventional *R*-factors *R* are based on *F*, with *F* set to zero for negative *F*^2^. The threshold expression of *F*^2^ > σ(*F*^2^) is used only for calculating *R*-factors(gt) *etc*. and is not relevant to the choice of reflections for refinement. *R*-factors based on *F*^2^ are statistically about twice as large as those based on *F*, and *R*- factors based on ALL data will be even larger.

### Fractional atomic coordinates and isotropic or equivalent isotropic displacement parameters (Å^2^)

**Table:**

	*x*	*y*	*z*	*U*_iso_*/*U*_eq_	Occ. (<1)
C29	0.8351 (7)	0.64681 (19)	1.3342 (2)	0.0602 (12)	0.478 (5)
H29	0.9494	0.6434	1.3210	0.072*	0.478 (5)
Cl1	0.7003 (8)	0.5922 (3)	1.2794 (4)	0.0895 (14)	0.478 (5)
Cl2	0.8176 (5)	0.6354 (2)	1.4489 (3)	0.0877 (9)	0.478 (5)
Cl3	0.7464 (5)	0.72058 (6)	1.31137 (11)	0.0718 (8)	0.478 (5)
Cl1'	0.7252 (7)	0.5906 (2)	1.2724 (3)	0.0838 (10)	0.522 (5)
Cl2'	0.8644 (6)	0.63370 (14)	1.4441 (2)	0.1015 (12)	0.522 (5)
Cl3'	0.8354 (4)	0.71700 (8)	1.30123 (8)	0.0640 (4)	0.522 (5)
C29'	0.7290 (6)	0.65719 (17)	1.3448 (2)	0.0557 (10)	0.522 (5)
H29'	0.6178	0.6701	1.3559	0.067*	0.522 (5)
O1	0.20448 (12)	0.62134 (4)	0.86461 (7)	0.0458 (2)	
O2	0.37739 (12)	0.64726 (5)	0.68945 (6)	0.0451 (2)	
O3	0.47972 (15)	0.69449 (5)	1.02209 (6)	0.0588 (3)	
O4	0.10068 (14)	0.75164 (5)	0.58373 (6)	0.0550 (3)	
O5	0.87057 (12)	0.47605 (5)	0.89164 (8)	0.0550 (3)	
O6	−0.29654 (12)	0.50568 (5)	0.60580 (7)	0.0505 (3)	
C1	0.36050 (16)	0.71245 (6)	0.87968 (9)	0.0390 (3)	
C2	0.42348 (18)	0.73704 (7)	0.95995 (9)	0.0447 (3)	
C3	0.41933 (19)	0.80154 (7)	0.97694 (10)	0.0515 (4)	
H3	0.4636	0.8175	1.0324	0.062*	
C4	0.35117 (19)	0.84034 (7)	0.91280 (10)	0.0502 (4)	
H4	0.3462	0.8837	0.9245	0.060*	
C5	0.28728 (17)	0.81810 (6)	0.82908 (10)	0.0438 (3)	
C6	0.22155 (19)	0.85978 (7)	0.76273 (11)	0.0501 (4)	
H6	0.2223	0.9032	0.7748	0.060*	
C7	0.15732 (19)	0.83947 (7)	0.68225 (10)	0.0497 (4)	
H7	0.1129	0.8683	0.6387	0.060*	
C8	0.15723 (18)	0.77515 (7)	0.66393 (9)	0.0433 (3)	
C9	0.22199 (16)	0.73223 (6)	0.72575 (9)	0.0382 (3)	
C10	0.28973 (16)	0.75288 (6)	0.81102 (9)	0.0375 (3)	
C11	0.34302 (17)	0.64259 (6)	0.87375 (8)	0.0386 (3)	
C12	0.48719 (17)	0.60144 (6)	0.87996 (8)	0.0384 (3)	
C13	0.64446 (17)	0.62349 (6)	0.87923 (9)	0.0423 (3)	
H13	0.6620	0.6671	0.8765	0.051*	
C14	0.77667 (17)	0.58352 (6)	0.88242 (9)	0.0419 (3)	
H14	0.8834	0.5995	0.8812	0.050*	
C15	0.75081 (17)	0.51999 (6)	0.88745 (9)	0.0427 (3)	
C16	0.59450 (19)	0.49700 (7)	0.88921 (12)	0.0541 (4)	
H16	0.5775	0.4534	0.8935	0.065*	
C17	0.46492 (19)	0.53710 (7)	0.88481 (11)	0.0499 (4)	
H17	0.3582	0.5209	0.8850	0.060*	
C18	0.23968 (17)	0.66618 (6)	0.69476 (8)	0.0381 (3)	
C19	0.09406 (16)	0.62645 (6)	0.67084 (8)	0.0379 (3)	
C20	−0.06117 (17)	0.64490 (6)	0.68346 (9)	0.0409 (3)	
H20	−0.0758	0.6851	0.7065	0.049*	
C21	−0.19562 (17)	0.60641 (6)	0.66341 (9)	0.0411 (3)	
H21	−0.3008	0.6198	0.6731	0.049*	
C22	−0.17404 (17)	0.54777 (6)	0.62890 (9)	0.0398 (3)	
C23	−0.01928 (18)	0.52839 (7)	0.61557 (10)	0.0482 (4)	
H23	−0.0047	0.4883	0.5921	0.058*	
C24	0.11261 (18)	0.56729 (7)	0.63632 (10)	0.0457 (3)	
H24	0.2179	0.5537	0.6270	0.055*	
C25	0.5735 (2)	0.71604 (9)	1.10090 (11)	0.0643 (5)	
H25A	0.5032	0.7400	1.1343	0.077*	
H25B	0.6624	0.7427	1.0871	0.077*	
H25C	0.6194	0.6803	1.1354	0.077*	
C26	0.0351 (2)	0.79353 (9)	0.51653 (11)	0.0641 (5)	
H26A	0.1155	0.8261	0.5102	0.077*	
H26B	−0.0645	0.8127	0.5316	0.077*	
H26C	0.0093	0.7707	0.4616	0.077*	
C27	1.03147 (19)	0.49649 (8)	0.88276 (13)	0.0560 (4)	
H27A	1.0710	0.5251	0.9303	0.067*	
H27B	1.0292	0.5179	0.8269	0.067*	
H27C	1.1045	0.4604	0.8850	0.067*	
C28	−0.45787 (18)	0.52379 (8)	0.61699 (12)	0.0555 (4)	
H28A	−0.4892	0.5611	0.5823	0.067*	
H28B	−0.4612	0.5328	0.6785	0.067*	
H28C	−0.5341	0.4899	0.5978	0.067*	

### Atomic displacement parameters (Å^2^)

**Table:**

	*U*^11^	*U*^22^	*U*^33^	*U*^12^	*U*^13^	*U*^23^
C29	0.063 (3)	0.065 (2)	0.051 (2)	0.0107 (19)	0.0063 (18)	−0.0035 (16)
Cl1	0.1159 (17)	0.062 (2)	0.091 (2)	−0.0248 (15)	0.0161 (12)	0.0046 (14)
Cl2	0.0914 (12)	0.124 (2)	0.0476 (9)	0.0204 (10)	0.0102 (7)	0.0316 (10)
Cl3	0.112 (2)	0.0424 (5)	0.0544 (6)	0.0055 (7)	−0.0110 (8)	0.0000 (4)
Cl1'	0.141 (3)	0.0423 (13)	0.0614 (10)	0.0083 (14)	−0.0076 (14)	−0.0034 (8)
Cl2'	0.179 (3)	0.0723 (11)	0.0468 (8)	0.0443 (15)	−0.0057 (15)	0.0061 (7)
Cl3'	0.0718 (11)	0.0636 (6)	0.0553 (5)	−0.0131 (6)	0.0053 (5)	0.0089 (4)
C29'	0.057 (3)	0.061 (2)	0.0502 (17)	0.0037 (17)	0.0137 (15)	0.0103 (14)
O1	0.0442 (6)	0.0391 (5)	0.0528 (6)	−0.0031 (4)	0.0031 (4)	0.0031 (4)
O2	0.0432 (6)	0.0435 (5)	0.0480 (5)	0.0026 (4)	0.0045 (4)	−0.0020 (4)
O3	0.0831 (8)	0.0482 (6)	0.0394 (5)	0.0039 (5)	−0.0101 (5)	−0.0024 (4)
O4	0.0731 (7)	0.0468 (6)	0.0411 (5)	0.0015 (5)	−0.0047 (5)	0.0081 (4)
O5	0.0428 (6)	0.0337 (5)	0.0893 (8)	−0.0004 (4)	0.0123 (5)	−0.0042 (5)
O6	0.0427 (6)	0.0380 (5)	0.0697 (7)	−0.0029 (4)	0.0048 (5)	−0.0082 (5)
C1	0.0404 (7)	0.0348 (7)	0.0411 (7)	−0.0010 (5)	0.0034 (5)	−0.0018 (5)
C2	0.0496 (8)	0.0418 (7)	0.0412 (7)	−0.0005 (6)	0.0018 (6)	−0.0010 (6)
C3	0.0581 (9)	0.0462 (8)	0.0482 (8)	−0.0056 (7)	0.0014 (7)	−0.0112 (7)
C4	0.0571 (9)	0.0341 (7)	0.0588 (9)	−0.0040 (6)	0.0069 (7)	−0.0082 (6)
C5	0.0438 (8)	0.0340 (7)	0.0532 (8)	−0.0024 (6)	0.0059 (6)	−0.0010 (6)
C6	0.0540 (9)	0.0300 (7)	0.0657 (10)	−0.0004 (6)	0.0062 (7)	0.0030 (6)
C7	0.0537 (9)	0.0360 (7)	0.0578 (9)	0.0019 (6)	0.0032 (7)	0.0117 (6)
C8	0.0444 (8)	0.0406 (7)	0.0439 (7)	−0.0011 (6)	0.0032 (6)	0.0055 (6)
C9	0.0394 (7)	0.0331 (7)	0.0419 (7)	−0.0020 (5)	0.0048 (5)	0.0019 (5)
C10	0.0363 (7)	0.0338 (7)	0.0423 (7)	−0.0018 (5)	0.0053 (5)	0.0000 (5)
C11	0.0457 (8)	0.0365 (7)	0.0325 (6)	−0.0017 (6)	0.0022 (5)	0.0017 (5)
C12	0.0450 (8)	0.0332 (6)	0.0358 (6)	−0.0026 (6)	0.0015 (5)	0.0006 (5)
C13	0.0493 (8)	0.0304 (6)	0.0455 (7)	−0.0033 (6)	0.0015 (6)	0.0019 (5)
C14	0.0416 (8)	0.0367 (7)	0.0463 (7)	−0.0055 (6)	0.0028 (6)	−0.0002 (6)
C15	0.0443 (8)	0.0344 (7)	0.0486 (8)	0.0002 (6)	0.0048 (6)	−0.0030 (6)
C16	0.0489 (9)	0.0300 (7)	0.0839 (11)	−0.0046 (6)	0.0114 (8)	0.0001 (7)
C17	0.0433 (8)	0.0369 (7)	0.0701 (10)	−0.0049 (6)	0.0096 (7)	0.0012 (7)
C18	0.0437 (8)	0.0369 (7)	0.0329 (6)	0.0018 (6)	0.0033 (5)	0.0032 (5)
C19	0.0419 (8)	0.0358 (7)	0.0349 (6)	0.0013 (5)	0.0018 (5)	0.0015 (5)
C20	0.0475 (8)	0.0331 (6)	0.0416 (7)	0.0041 (6)	0.0048 (6)	−0.0037 (5)
C21	0.0419 (8)	0.0391 (7)	0.0421 (7)	0.0035 (6)	0.0051 (6)	−0.0009 (6)
C22	0.0436 (8)	0.0344 (7)	0.0403 (7)	−0.0008 (6)	0.0019 (5)	0.0019 (5)
C23	0.0485 (9)	0.0349 (7)	0.0604 (9)	0.0022 (6)	0.0056 (7)	−0.0102 (6)
C24	0.0420 (8)	0.0396 (7)	0.0550 (8)	0.0048 (6)	0.0056 (6)	−0.0053 (6)
C25	0.0759 (12)	0.0688 (11)	0.0424 (8)	0.0096 (9)	−0.0110 (8)	−0.0070 (8)
C26	0.0756 (12)	0.0637 (10)	0.0486 (9)	0.0055 (9)	−0.0050 (8)	0.0185 (8)
C27	0.0432 (9)	0.0447 (8)	0.0808 (11)	−0.0007 (6)	0.0118 (8)	−0.0036 (8)
C28	0.0440 (9)	0.0486 (9)	0.0738 (11)	−0.0047 (7)	0.0081 (7)	−0.0076 (8)

### Geometric parameters (Å, º)

**Table:**

C29—Cl1	1.751 (7)	C11—C12	1.4769 (19)
C29—Cl3	1.761 (5)	C12—C13	1.387 (2)
C29—Cl2	1.824 (6)	C12—C17	1.3975 (19)
C29—H29	1.0000	C13—C14	1.386 (2)
Cl1'—C29'	1.816 (6)	C13—H13	0.9500
Cl2'—C29'	1.840 (6)	C14—C15	1.3852 (19)
Cl3'—C29'	1.749 (4)	C14—H14	0.9500
C29'—H29'	1.0000	C15—C16	1.389 (2)
O1—C11	1.2230 (17)	C16—C17	1.369 (2)
O2—C18	1.2243 (16)	C16—H16	0.9500
O3—C2	1.3608 (17)	C17—H17	0.9500
O3—C25	1.4273 (18)	C18—C19	1.4791 (19)
O4—C8	1.3621 (17)	C19—C20	1.3859 (19)
O4—C26	1.4238 (18)	C19—C24	1.3964 (19)
O5—C15	1.3633 (17)	C20—C21	1.385 (2)
O5—C27	1.4282 (18)	C20—H20	0.9500
O6—C22	1.3665 (16)	C21—C22	1.3906 (19)
O6—C28	1.4260 (18)	C21—H21	0.9500
C1—C2	1.3839 (19)	C22—C23	1.391 (2)
C1—C10	1.4323 (18)	C23—C24	1.375 (2)
C1—C11	1.5087 (19)	C23—H23	0.9500
C2—C3	1.412 (2)	C24—H24	0.9500
C3—C4	1.357 (2)	C25—H25A	0.9800
C3—H3	0.9500	C25—H25B	0.9800
C4—C5	1.414 (2)	C25—H25C	0.9800
C4—H4	0.9500	C26—H26A	0.9800
C5—C6	1.413 (2)	C26—H26B	0.9800
C5—C10	1.4291 (19)	C26—H26C	0.9800
C6—C7	1.357 (2)	C27—H27A	0.9800
C6—H6	0.9500	C27—H27B	0.9800
C7—C8	1.410 (2)	C27—H27C	0.9800
C7—H7	0.9500	C28—H28A	0.9800
C8—C9	1.3814 (19)	C28—H28B	0.9800
C9—C10	1.4311 (18)	C28—H28C	0.9800
C9—C18	1.5122 (18)		
			
Cl1—C29—Cl3	106.7 (4)	C15—C14—H14	120.5
Cl1—C29—Cl2	104.4 (3)	C13—C14—H14	120.5
Cl3—C29—Cl2	103.2 (3)	O5—C15—C14	124.55 (13)
Cl1—C29—H29	113.8	O5—C15—C16	115.23 (12)
Cl3—C29—H29	113.8	C14—C15—C16	120.22 (13)
Cl2—C29—H29	113.8	C17—C16—C15	120.05 (13)
Cl3'—C29'—Cl1'	107.6 (3)	C17—C16—H16	120.0
Cl3'—C29'—Cl2'	104.3 (3)	C15—C16—H16	120.0
Cl1'—C29'—Cl2'	104.8 (3)	C16—C17—C12	121.05 (14)
Cl3'—C29'—H29'	113.1	C16—C17—H17	119.5
Cl1'—C29'—H29'	113.1	C12—C17—H17	119.5
Cl2'—C29'—H29'	113.1	O2—C18—C19	121.62 (12)
C2—O3—C25	118.50 (13)	O2—C18—C9	117.87 (12)
C8—O4—C26	118.65 (13)	C19—C18—C9	120.51 (12)
C15—O5—C27	117.69 (11)	C20—C19—C24	118.10 (13)
C22—O6—C28	117.37 (11)	C20—C19—C18	122.55 (12)
C2—C1—C10	119.91 (12)	C24—C19—C18	119.33 (13)
C2—C1—C11	117.03 (12)	C21—C20—C19	121.93 (12)
C10—C1—C11	122.17 (12)	C21—C20—H20	119.0
O3—C2—C1	115.31 (12)	C19—C20—H20	119.0
O3—C2—C3	122.79 (13)	C20—C21—C22	118.88 (13)
C1—C2—C3	121.78 (13)	C20—C21—H21	120.6
C4—C3—C2	118.97 (14)	C22—C21—H21	120.6
C4—C3—H3	120.5	O6—C22—C21	124.61 (13)
C2—C3—H3	120.5	O6—C22—C23	115.29 (12)
C3—C4—C5	121.83 (14)	C21—C22—C23	120.10 (13)
C3—C4—H4	119.1	C24—C23—C22	120.02 (13)
C5—C4—H4	119.1	C24—C23—H23	120.0
C6—C5—C4	120.62 (13)	C22—C23—H23	120.0
C6—C5—C10	119.53 (13)	C23—C24—C19	120.97 (14)
C4—C5—C10	119.85 (13)	C23—C24—H24	119.5
C7—C6—C5	121.71 (14)	C19—C24—H24	119.5
C7—C6—H6	119.1	O3—C25—H25A	109.5
C5—C6—H6	119.1	O3—C25—H25B	109.5
C6—C7—C8	119.29 (13)	H25A—C25—H25B	109.5
C6—C7—H7	120.4	O3—C25—H25C	109.5
C8—C7—H7	120.4	H25A—C25—H25C	109.5
O4—C8—C9	115.78 (12)	H25B—C25—H25C	109.5
O4—C8—C7	122.51 (13)	O4—C26—H26A	109.5
C9—C8—C7	121.64 (13)	O4—C26—H26B	109.5
C8—C9—C10	119.75 (12)	H26A—C26—H26B	109.5
C8—C9—C18	116.77 (12)	O4—C26—H26C	109.5
C10—C9—C18	122.85 (11)	H26A—C26—H26C	109.5
C5—C10—C9	118.06 (12)	H26B—C26—H26C	109.5
C5—C10—C1	117.65 (12)	O5—C27—H27A	109.5
C9—C10—C1	124.29 (12)	O5—C27—H27B	109.5
O1—C11—C12	121.28 (12)	H27A—C27—H27B	109.5
O1—C11—C1	117.27 (12)	O5—C27—H27C	109.5
C12—C11—C1	121.45 (12)	H27A—C27—H27C	109.5
C13—C12—C17	118.00 (13)	H27B—C27—H27C	109.5
C13—C12—C11	123.07 (12)	O6—C28—H28A	109.5
C17—C12—C11	118.91 (13)	O6—C28—H28B	109.5
C14—C13—C12	121.69 (12)	H28A—C28—H28B	109.5
C14—C13—H13	119.2	O6—C28—H28C	109.5
C12—C13—H13	119.2	H28A—C28—H28C	109.5
C15—C14—C13	118.97 (13)	H28B—C28—H28C	109.5
			
C25—O3—C2—C1	168.44 (14)	C10—C1—C11—C12	121.00 (14)
C25—O3—C2—C3	−15.5 (2)	O1—C11—C12—C13	171.55 (13)
C10—C1—C2—O3	176.54 (12)	C1—C11—C12—C13	−9.31 (19)
C11—C1—C2—O3	7.2 (2)	O1—C11—C12—C17	−6.9 (2)
C10—C1—C2—C3	0.4 (2)	C1—C11—C12—C17	172.21 (13)
C11—C1—C2—C3	−168.95 (14)	C17—C12—C13—C14	0.5 (2)
O3—C2—C3—C4	−175.58 (15)	C11—C12—C13—C14	−177.95 (12)
C1—C2—C3—C4	0.2 (2)	C12—C13—C14—C15	−0.7 (2)
C2—C3—C4—C5	−1.3 (2)	C27—O5—C15—C14	−5.6 (2)
C3—C4—C5—C6	−178.04 (14)	C27—O5—C15—C16	174.88 (14)
C3—C4—C5—C10	1.7 (2)	C13—C14—C15—O5	−179.46 (13)
C4—C5—C6—C7	−179.00 (15)	C13—C14—C15—C16	0.0 (2)
C10—C5—C6—C7	1.3 (2)	O5—C15—C16—C17	−179.60 (15)
C5—C6—C7—C8	−0.5 (2)	C14—C15—C16—C17	0.9 (2)
C26—O4—C8—C9	−178.84 (14)	C15—C16—C17—C12	−1.1 (3)
C26—O4—C8—C7	−1.7 (2)	C13—C12—C17—C16	0.4 (2)
C6—C7—C8—O4	−177.39 (14)	C11—C12—C17—C16	178.92 (15)
C6—C7—C8—C9	−0.5 (2)	C8—C9—C18—O2	107.25 (15)
O4—C8—C9—C10	177.78 (12)	C10—C9—C18—O2	−63.58 (17)
C7—C8—C9—C10	0.6 (2)	C8—C9—C18—C19	−72.04 (17)
O4—C8—C9—C18	6.65 (19)	C10—C9—C18—C19	117.13 (14)
C7—C8—C9—C18	−170.49 (13)	O2—C18—C19—C20	174.23 (12)
C6—C5—C10—C9	−1.1 (2)	C9—C18—C19—C20	−6.50 (19)
C4—C5—C10—C9	179.24 (13)	O2—C18—C19—C24	−3.99 (19)
C6—C5—C10—C1	178.76 (13)	C9—C18—C19—C24	175.27 (12)
C4—C5—C10—C1	−0.9 (2)	C24—C19—C20—C21	0.4 (2)
C8—C9—C10—C5	0.1 (2)	C18—C19—C20—C21	−177.84 (12)
C18—C9—C10—C5	170.69 (13)	C19—C20—C21—C22	−0.6 (2)
C8—C9—C10—C1	−179.69 (13)	C28—O6—C22—C21	0.9 (2)
C18—C9—C10—C1	−9.1 (2)	C28—O6—C22—C23	−179.05 (14)
C2—C1—C10—C5	−0.1 (2)	C20—C21—C22—O6	−179.47 (13)
C11—C1—C10—C5	168.75 (13)	C20—C21—C22—C23	0.4 (2)
C2—C1—C10—C9	179.75 (13)	O6—C22—C23—C24	179.76 (14)
C11—C1—C10—C9	−11.4 (2)	C21—C22—C23—C24	−0.2 (2)
C2—C1—C11—O1	109.29 (15)	C22—C23—C24—C19	0.0 (2)
C10—C1—C11—O1	−59.83 (18)	C20—C19—C24—C23	−0.1 (2)
C2—C1—C11—C12	−69.88 (17)	C18—C19—C24—C23	178.19 (13)

### Hydrogen-bond geometry (Å, º)

**Table:**

*D*—H···*A*	*D*—H	H···*A*	*D*···*A*	*D*—H···*A*
C7—H7···O5^i^	0.95	2.37	3.1460 (19)	139
C13—H13···Cl3^ii^	0.95	2.75	3.647 (2)	159

Symmetry codes: (i) −*x*+1, *y*+1/2, −*z*+3/2; (ii) *x*, −*y*+3/2, *z*−1/2.

## References

[bb1] Burnett, M. N. & Johnson, C. K. (1996). *ORTEPIII* Report ORNL-6895. Oak Ridgeational Laboratory, Tennessee, USA.

[bb2] Higashi, T. (1999). *NUMABS* Rigaku Corporation, Tokyo, Japan.

[bb3] Hijikata, D., Takada, T., Nagasawa, A., Okamoto, A. & Yonezawa, N. (2010). *Acta Cryst.* E**66**, o2902–o2903. 10.1107/S1600536810042170 PMC3009209 21589079

[bb4] Okamoto, A., Mitsui, R., Oike, H. & Yonezawa, N. (2011). *Chem* *Lett* **40**, 1283–1284.

[bb5] Okamoto, A. & Yonezawa, N. (2009). *Chem. Lett.* **38**, 914–915.

[bb6] Rigaku (1998). *PROCESS-AUTO* Rigaku Corporation, Tokyo, Japan.

[bb7] Rigaku (2010). *CrystalStructure* Rigaku Corporation, Tokyo, Japan.

[bb8] Sasagawa, K., Muto, T., Okamoto, A., Oike, H. & Yonezawa, N. (2011). *Acta Cryst.* E**67**, o3354. 10.1107/S1600536811048550 PMC3238999 22199848

[bb9] Sheldrick, G. M. (2008). *Acta Cryst.* A**64**, 112–122. 10.1107/S0108767307043930 18156677

